# Cardiac mass many years after heart transplantation—a case report

**DOI:** 10.1007/s12055-022-01362-x

**Published:** 2022-05-27

**Authors:** Lucia Dallapellegrina, Edoardo Sciatti, Enrico Vizzardi, Antonio D’Aloia

**Affiliations:** 1 Cardiology Unit, Department of Medical and Surgical Specialties, Radiological Sciences and Public Health, University of Brescia, Brescia, Italy; 2 Cardio-Thoracic Department, ASST Spedali Civili, Brescia, Italy; 3 Cardiology Unit 1, Cardiovascular Department, ASST Papa Giovanni XXIII, Piazza OMS 1, 24127 Bergamo, Italy

**Keywords:** Heart transplantation, Bi-caval technique, thrombus, Giant neo-atrium, Bi-atrial technique

## Abstract

The bi-atrial surgical technique of heart transplantation is associated with postoperative atrial dysfunction, sinus node dysfunction, valvular dysfunction, and bi-atrial enlargement predisposing to atrial arrhythmia with thrombus formation. This report deals with a very late thrombus formation in the neo-atrium of a heart transplanted using the bi-atrial technique. The absence of arrhythmia and absence of any history of intake of prothrombotic medications make it noteworthy.

**Supplementary Information:**

The online version contains supplementary material available at 10.1007/s12055-022-01362-x.

## Introduction

The bi-atrial surgical technique of heart transplantation is one of the standard options for orthotopic heart transplants. This technique takes less time than the bi-caval technique, which is, however, now more commonly practiced than the bi-atrial one since the latter has been associated with postoperative problems including atrial dysfunction, sinus node dysfunction, valvular dysfunction, and bi-atrial enlargement predisposing to atrial arrhythmia with thrombus formation. The precise incidence of bi-atrial thrombus formation after orthotopic heart transplantation is not clear, and only few cases have been reported in the literature.

## Case report

**Fig. 1:**
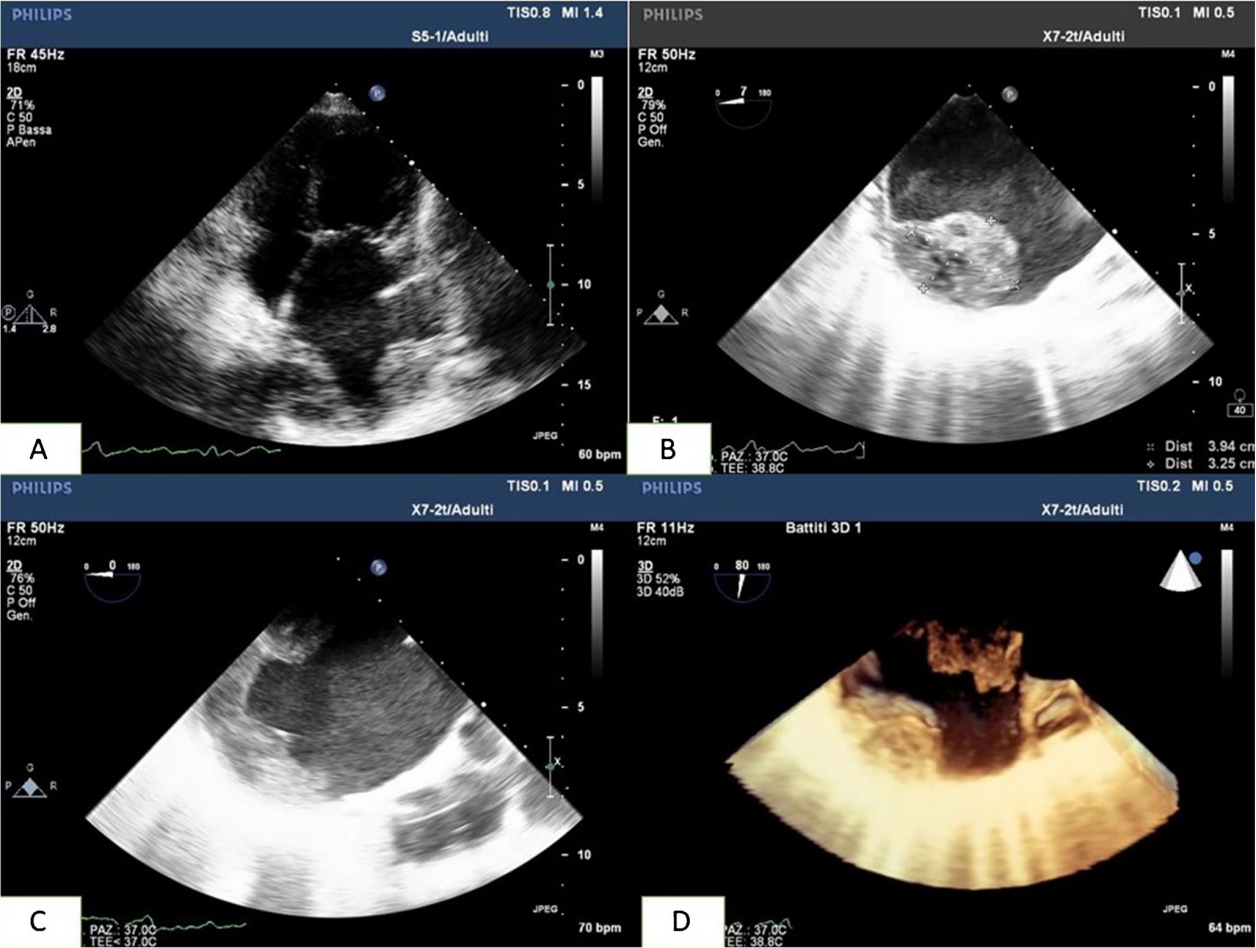
Transthoracic (**A**) and transesophageal (**B**, **C**, **D**) echocardiography showing left atrial thrombotic formation

This case deals with a 73-year-old woman who underwent an orthotopic heart transplantation using the bi-atrial technique. The transplant was performed 17 years earlier for advanced heart failure due to idiopathic dilated cardiomyopathy. After the heart transplantation, the patient was treated with cyclosporine, mycofenolate, bisoprolol, doxazosin, furosemide, acetylsalicylic acid, esomeprazole, and allopurinol. Her clinical history was characterized, 10 years after transplant, by an episode of graft rejection grade 1R requiring steroid administration, by the need for a percutaneous coronary intervention with drug-eluting stent implantation at the proximal segment of the anterior descending artery, and then 3 years later by paroxysmal total heart block associated with syncope requiring permanent bicameral pacemaker implantation. She was also affected by systemic arterial hypertension, iatrogenic (steroid) diabetes mellitus, renal failure, and hyperuricemia. In January 2020, the patient was admitted to the General Surgery Unit for surgical treatment of a sigmoid colon perforation following a complicated endoscopic polypectomy, and underwent laparoscopic bowel suturing and temporary ileostomy. The peri-operative cardiologic evaluation with echocardiography described normal left ventricular geometry and systolic function with grade II diastolic dysfunction, a dilated right ventricle with preserved pump function and mild pulmonary hypertension (estimated systolic pulmonary hypertension 45 mmHg), an extremely dilated left atrium (area 50 cm^2^), moderate mitral and aortic regurgitations, and a normal inferior vena cava. She remained pauci-symptomatic (New York Heart Association (NYHA) functional class II) until July 2020, when she attended the yearly cardiologic follow-up. A transthoracic echocardiography showed a huge extended neo-left atrium (area 67 cm^2^, volume 360 mL) with evidence of spontaneous echo contrast and intracavitary thrombus (Fig. 1A), normal left ventricular dimension (end-diastolic diameter 42 mm and volume 60 mL) and systolic function with grade II diastolic dysfunction, moderate (++) mitral regurgitation due to leaflets fibrosis, moderate (++) aortic regurgitation due to cusps fibrosclerosis, a dilated right ventricle and right atrium (area 27 cm^2^, volume 67 mL), severe pulmonary hypertension (estimated systolic pulmonary artery pressure 60 mmHg), and a mildly dilated inferior vena cava. A transesophageal echocardiogram was then performed, confirming the presence of a double heterogeneous formation localized onto the left atrial posterior wall, and principally extended to the septal and lateral walls (thickness 2.8–3 cm), with a small pedunculated component and not involving the four pulmonary veins’ ostia (Fig. 1B–D; Videos 1, 2, 3). These findings were interpreted as intracavitary thrombi. In addition, another thrombus was described at the left atrial appendage apex (thickness 1.2 cm).

**Fig. 2:**
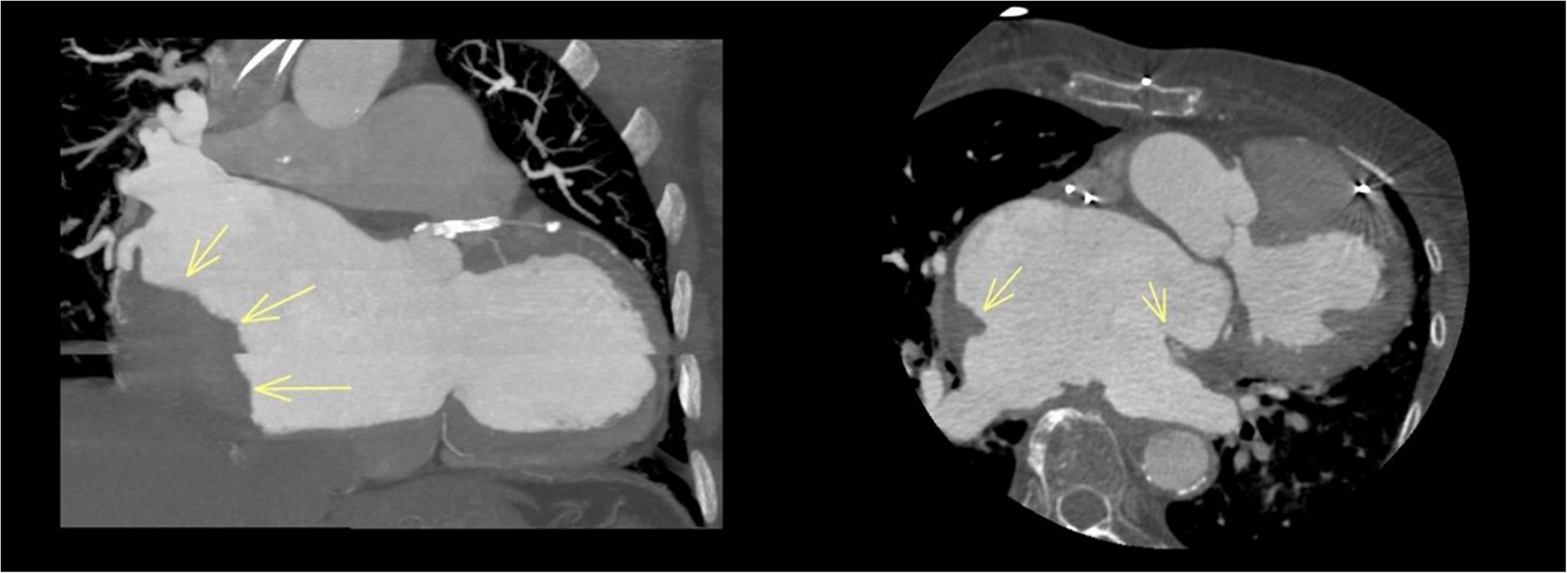
CT scan evidencing the thrombus in the neo-atrium

A cardiac computed tomography (CT) scan study was therefore performed, confirming the presence of a giant thrombotic formation in the left neo-atrium, posteriorly to the suture lines (Fig. 2). At pacemaker interrogation, no events of atrial arrhythmias were documented ever. The patient was treated with subcutaneous enoxaparin and then with oral anticoagulant (warfarin). One year later, the thrombus was still present, but reduced and stratified, with improvement of pulmonary hypertension and inferior vena cava dilation. Consequently, quality of life returned to what it was in 2020 and functional class improved to NYHA II.

## Discussion

Atrial masses are a rare complication in heart transplant recipients. They are usually diagnosed accidentally on routine transthoracic echocardiography, and other imaging techniques should be performed to assess the masses’ size, morphology, and precise localization. Standard orthotopic heart transplantation produces important anatomic and functional atrial alterations, with subsequent thrombotic risk. Left atrial thrombosis in heart transplant recipients is not a rare finding, particularly with the bi-atrial rather than the bi-caval technique, because larger neo-atria predispose also to supraventricular arrhythmias [1–4]. The incidence of atrial thrombi and spontaneous echocardiographic contrast after standard orthotopic (bi-atrial) heart transplantation was found to be 19–28% and 52–55% [2, 5], respectively, and the major risk factor for thrombus formation was, discordantly, atrial dilatation or low cardiac output [1, 6]. The suture line was reported to be a potential nidus for thrombus formation [7]. However, in our patient, arrhythmias have never been documented and we can hypothesize that colonic surgery was the *primum movens* of thrombus formation after 17 years from heart transplant. There were no symptoms or thromboembolic events in the past. In literature, most of the other cases reported were identified early after heart transplantation (mean 3–31 months) [2, 4] and only one case reported this finding after 14 years [8].

The main pathophysiological issues to be considered are the components of the Virchow’s triad, mostly referring to abnormal blood flow due to massive chamber dilation caused by the orthotopic technique, atrial scars following cardiac surgery, and a prothrombotic state related to abdominal surgery. Conventional cardiac transplantation using the bi-atrial technique alters atrial integrity, geometry, and possibly function. This can lead to enlarged atria, which can be hemodynamically impaired despite the presence of a sinus rhythm. Differently from some previous reports, the novelty of this case relates to the absence of other known factors favoring the development of cardiac thrombosis and the long time frame from heart transplantation. The patient maintained a constant sinus rhythm and was not on mammalian target of rapamycin (mTOR) inhibitors as immunosuppressant, which have prothrombotic properties. Accordingly, the novelty of this case relates to the knowledge of a reliable cause for the thrombotic phenomenon, namely the surgical intervention, given that the thrombus had never been reported before it.

## Supplementary information

Video 1 Transoesophageal echocardiography showing left atrial thrombotic formation. (MP4 2659 kb)

Video 2 Transoesophageal echocardiography showing left atrial thrombotic formation. (MP4 2634 kb)

Video 3 3D transoesophageal echocardiography showing left atrial thrombotic formation. (MP4 244 kb)
